# Detection of neutrophil extracellular traps in patient plasma: method development and validation in systemic lupus erythematosus and healthy donors that carry *IRF5* genetic risk

**DOI:** 10.3389/fimmu.2022.951254

**Published:** 2022-07-26

**Authors:** Bharati Matta, Jenna Battaglia, Betsy J. Barnes

**Affiliations:** ^1^ Center for Autoimmune Musculoskeletal and Hematopoietic Diseases, Feinstein Institutes for Medical Research, Manhasset, NY, United States; ^2^ Departments of Molecular Medicine and Pediatrics, Zucker School of Medicine at Hofstra/Northwell, Hempstead, NY, United States

**Keywords:** NETosis, ELISA, smear assay, immunofluorescence, quantification

## Abstract

Neutrophil extracellular traps (NETs) are web-like structures extruded by neutrophils after activation or in response to microorganisms. These extracellular structures are decondensed chromatin fibers loaded with antimicrobial granular proteins, peptides, and enzymes. NETs clear microorganisms, thus keeping a check on infections at an early stage, but if dysregulated, may be self-destructive to the body. Indeed, NETs have been associated with autoimmune diseases such as systemic lupus erythematosus (SLE), rheumatoid arthritis (RA), antiphospholipid syndrome (APS), psoriasis, and gout. More recently, increased NETs associate with COVID-19 disease severity. While there are rigorous and reliable methods to quantify NETs from neutrophils *via* flow cytometry and immunofluorescence, the accurate quantification of NETs in patient plasma or serum remains a challenge. Here, we developed new methodologies for the quantification of NETs in patient plasma using multiplex ELISA and immunofluorescence methodology. Plasma from patients with SLE, non-genotyped healthy controls, and genotyped healthy controls that carry either the homozygous risk or non-risk *IRF5*-SLE haplotype were used in this study. The multiplex ELISA using antibodies detecting myeloperoxidase (MPO), citrullinated histone H3 (CitH3) and DNA provided reliable detection of NETs in plasma samples from SLE patients and healthy donors that carry *IRF5* genetic risk. An immunofluorescence smear assay that utilizes only 1 µl of patient plasma provided similar results and data correlate to multiplex ELISA findings. The immunofluorescence smear assay is a relatively simple, inexpensive, and quantifiable method of NET detection for small volumes of patient plasma.

## Introduction

As the largest constituent of circulating white blood cells, neutrophils migrate to infected tissues in response to inflammatory stimuli where they protect the host by phagocytosing, killing, and digesting pathogens (1). Neutrophils are composed of many granules containing a variety of antimicrobial proteins. Hence, they have several strategies to kill microbes; one of them is through the release of neutrophil extracellular traps (NETs) (2). NETs are extracellular structures of decondensed chromatin fibers with attached neutrophil granular proteins, such as neutrophil elastase (NE), myeloperoxidase (MPO) and cathepsin G (3). NET formation involves the citrullination of histones by peptidyl arginine deiminase 4 (PAD4), chromatin decondensation, and disintegration of the nuclear membrane (4). Although this process, called NETosis, helps to protect the host from pathogens, when exaggerated or NET clearance is diminished, can contribute to tissue damage and autoimmunity (5). Hence, NETosis can be a double-edged sword and has been increasingly linked to the pathogenesis of multiple diseases, including systemic lupus erythematosus (SLE) (6, 7), rheumatoid arthritis (RA) (8), thrombosis (9), tuberculosis (TB) (10), vasculitis (11), gout (12, 13), diabetes (14), asthma-COPD (15, 16), and Coronavirus disease 2019 (COVID19) (17–19). Experimentally, NETs can be detected in 50µl undiluted plasma samples using single anti-MPO, anti-NE, or anti-Citrullinated H3 (CitH3) as capture antibodies with anti-DNA as the detection antibody in ELISA (20, 21). Data are generally presented as absorbance values, percentage of healthy adult plasma set at 100%, or as quantified DNA content using an in-house generated standard (17, 22). In this study, we developed and optimized a sensitive multiplex ELISA, as well as designed a simple, new immunofluorescence technique to both visualize and quantify NETs in small amounts of patient plasma. To validate these methods, we quantified plasma NETs in SLE patients and healthy controls that carry *IRF5* genetic risk to assess the range and sensitivity of NET detection by these two assays.

SLE is a complex, heterogeneous autoimmune disease characterized by high levels of autoantibodies. It is now well-accepted that NETs are a source of autoantigen leading to the production of type I IFNs and SLE autoantibodies, with higher levels of NETs and/or decreased NET clearance associating with disease pathogenesis (23). *IRF5* genetic variants have the second highest odds ratio for SLE risk, and the identification of four SNPs within the regulatory regions of *IRF5*, when carried on both alleles, are highly associated with SLE risk and are defined as the homozygous *IRF5*-SLE risk haplotype (24). We recently reported that healthy individuals carrying this haplotype are more susceptible to environmental and stochastic influences that trigger chronic immune activation, predisposing to the development of clinical SLE (25). In particular, we found that neutrophils from homozygous risk versus non-risk healthy individuals underwent increased spontaneous NETosis, and these NETs could activate plasmacytoid dendritic cells (pDCs), leading to the differentiation of naïve B cells to antibody-producing plasma cells (25). Indeed, work from numerous labs now support the critical role that NETs play in bridging innate and adaptive immunity (23). Here, we established a more sensitive ELISA using two capture antibodies and a novel immunofluorescence smear technique to visualize NETs in SLE patients and genotyped healthy controls. We further developed a method to quantify NETs from immunofluorescence smear assay using ImageJ software.

## Materials, equipment, and methods

### Human samples

All experiments were approved by the Feinstein Institutes for Medical Research IRB. Informed consent was obtained from all healthy donors and SLE patients prior to inclusion in the study, and experiments were performed in accordance with institutional and regional guidelines. Each of the patients fulfilled at least four of the classification criteria for SLE as defined by the American College of Rheumatology (ACR).

### Induction of NETs in human plasma

Peripheral blood (20mL) was collected in EDTA tubes from non-genotyped healthy donors (n=7). 10mL of whole blood was left unstimulated, and 10mL was stimulated with 100nM PMA (Sigma-Aldrich #P8139) for 2 hours at 37°C. Samples were centrifuged at 3000 RPM for 10 minutes and plasma was collected into small aliquots and stored at -80°C.

### SLE and GaP samples

Blood samples (n=40) from SLE patients (male and female) were obtained from the rheumatology clinic at Northwell Health. Blood samples from age- and gender-matched healthy donors served as the controls for SLE patients. Plasma was collected and stored at -80°C. Blood samples from healthy donors carrying the homozygous *IRF5*-SLE risk (n=10) and non-risk (n=12) haplotype were obtained from the Genotype and Phenotype (GaP) registry (26). The GaP registry samples are genotyped on the Illumina Human Immunochip and donors were selected as previously described (25, 26).

### Sytox green assay

50µl of 10x diluted plasma sample was added to black 96-well microplate (Greiner Bio-One). Sytox Green fluorescent dye at a concentration of 5µM (ThermoFisher #S33025) was added to each well. The plate was incubated in the dark for 5 minutes and fluorescence was read on a Synergy Neo2 Multi-Mode Microplate Reader (BioTek) with excitation and emission wavelengths of 485/527.

### ELISA

To detect circulating NETs in plasma, 96-well ultra-high binding plates (Thermo Scientific Ultra High Binding Polystyrene Microtiter) were coated with either 50µl of anti-NE(G2) antibody (SantaCruz Biotechnology #55549), anti-MPO(4A4/2C7) antibody (BioRad#0400-0002, or Abcam #25989), or anti-CitH3(R2 R8 R17) antibody (Abcam#5103) diluted to 5µg/mL in coating buffer (15mM Na_2_CO_3_, 35mM NaHCO_3_, pH 9.6) and incubated at 4°C overnight. In some experiments, plates were coated with a combination of anti-MPO(Abcam) and -CitH3(Abcam) antibodies at a concentration of 5µg/mL each. The next morning, the wells were washed three times with 200µL PBS. After the last wash, the plate was blocked with 200µl blocking buffer (5% BSA/PBS) for 2 hours at room temperature. For all anti-MPO and -CitH3 antibodies, 5% Normal Rat Serum (Fisher Scientific10-710-C) was added to the blocking buffer. The plate was then washed five times with PBS and left to incubate for 2 hours with 50µl of undiluted plasma sample. After washing the plate five times with wash buffer (1% BSA/PBS + 0.05% Tween20), 50µl of anti-DNA antibody (Roche-SigmaAldrich, Cell Death Detection ELISAPLUS Anti-DNA POD #11774425001) diluted 1:100 in 5%BSA + 0.05% Tween20 was added to the plate and incubated for 2 hours. The plate was washed five times with the previously mentioned wash buffer before adding 100µl TMB (3,3’,5,5’-tetramethylbenzidine, Southern Biotech #0410-01), allowed to come to room temperature before using. The plate was left in the dark to develop for 3 minutes before 100µl of stop solution (2N Sulfuric Acid, Reagents #CS106300-500A) was added. Absorbance of the plate was measured using a Synergy Neo2 Multi-Mode Microplate Reader (BioTek) at a wavelength of 450nm.

### Preparing in-house standard for the quantification of circulating NETs by ELISA

For standard curve preparation, three PMA-stimulated healthy donor plasma samples were combined, and two times serial dilution of plasma was made using PBS + 0.5M EDTA. DNA was quantified using Nanodrop (ThermoFisher). Serially diluted PMA-induced plasma samples were used as standards in duplicate with CitH3 and MPO+CitH3 ELISA methodology. Four-parameter logistic standard curves were created for each antibody coating and data was extrapolated using My Assays online software (27).

### Plasma NET smear assay

Poly-L-Lysine glass slides (Newcomer Supply #5010) were prepared for each plasma sample by marking the slide with a small circle using a histology liquid repellent pen. 1µl of the plasma sample was added to each circle and smeared in a circular motion until the volume was equally distributed. The samples were fixed with 100µl of 4% Formaldehyde for 10 minutes. The slides were tilted to remove the liquid on a paper towel, and each circle was carefully washed three times with PBS. After washing, the slides were blocked with 5%BSA/PBS for 1 hour and then stained with Sytox Green nucleic acid stain diluted 1:200 (Invitrogen #S7020) and 5 mg/mL DAPI (BioLegend #422801) for 15 minutes. After a final 3 washes, the coverslip was secured with VectaMount AQ Mounting Medium (Vector Labs #H-5501) and the slides dried before imaging. Images were taken at 2x and 20x using Invitrogen EVOS M7000 Imaging System and ZEISS Confocal M880. In a similar fashion, after blocking and washing, slides were incubated with anti-MPO (Abcam#25989) and -CitH3 (Abcam#5103) antibodies diluted to 1µg/mL in 0.3% Tritonx100 + 0.1%BSA solution overnight. The following morning, slides were washed three times with PBS and then incubated with goat anti-mouse AlexaFluor488 (IgG(H+L), 2mg/mL, Invitrogen #A11029) and goat anti-rabbit AlexaFluor594 (IgG(H+L), 2mg/mL, Molecular Probes #A11012) secondary antibodies diluted 1:500 for 1 hour. After washing three additional times with PBS, slides were stained with 5mg/mL DAPI (BioLegend) for 15 minutes and then imaged as described above.

### NET quantification using Image

Images of NET-smeared slides were taken at 20x using ZEISS Confocal M880. Representative images were analyzed in ImageJ Java-based program (V1.8) and converted to grayscale 8-bit images. Measurements were set to be limited to threshold, and threshold limits were set for each image in a pixel intensity range of 40(min) to 255 (max). Images were then individually measured to report an average intensity of the threshold area, representing circulating NETs. Averages were taken for each sample group including healthy controls, PMA-stimulated, SLE patient, and GaP samples using ImageJ result summarizing features (28). In addition, the same selected slides were imaged at 2x using Invitrogen EVOS M7000 Imaging System and NETs quantified using the same pixel intensity threshold limits of 40-255 to compare the area of threshold intensity between the two objectives.

### Statistical analysis

All statistical analyses were performed and graphed using GraphPad Prism 8. A two-tailed parametric *t* test was used for comparisons between samples with normal distribution. *P*<0.05 was considered statistically significant. A two-tailed correlation analysis was preformed using Pearson correlation coefficients assuming Gaussian distribution.

## Results

### Sytox green assay

**Figure 1:**
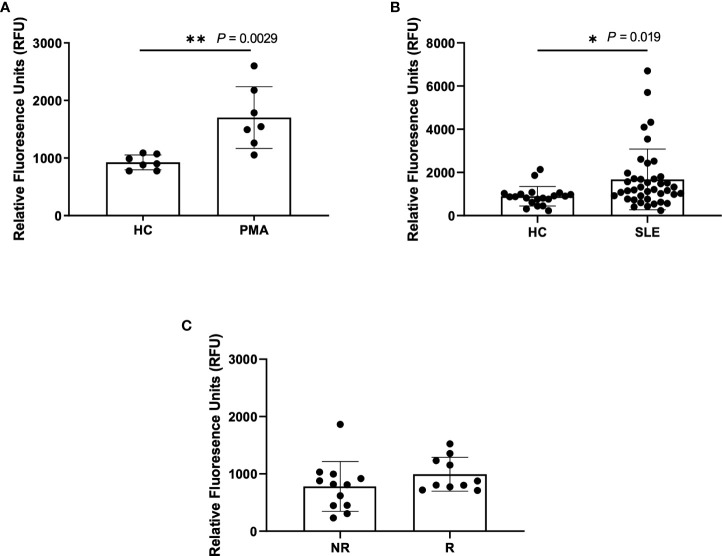
Higher NETs are detected in plasma from PMA treated samples and SLE samples using Sytox Green assay. Plasma samples were incubated with Sytox Green for 5 minutes and fluorescence was measured using microplate reader. **(A)** Plasma samples from untreated healthy controls (HC) and PMA treated healthy donors (PMA, n=7), **(B)** SLE patients (n=40) and healthy controls (n=20), and **(C)** non-risk (NR, n=12) and risk (R, n=10) donors from the Genotype and Phenotype Registry. Single data points represent individual donors. Plotted data are after background subtraction. Data are presented as mean ± SD. P values are reported after unpaired parametric T test was performed *<0.05; **<0.01.

A standard, well-accepted method of NET detection directly from neutrophils is the staining of extracellular DNA with Sytox Green (29–33). This technique is also used to assess NETs in patient plasma/serum yet is not specific for NETosis alone. Using this method, we detected a significant increase in DNA content within the plasma of PMA-stimulated healthy blood samples (n=7) as compared to untreated healthy controls (n=7), as expected (**Figure 1A**). Similarly, we detected a significant increase in DNA content within SLE plasma (**Figure 1B**). Given our previous finding of increased spontaneous NETosis and anti-MPO autoantibodies from healthy donors that carry *IRF5* homozygous risk (25), we were somewhat surprised to only find a trend of increased DNA content in homozygous risk versus non-risk samples that was not statistically significant (**Figure 1C**).

### ELISA

Another standard method of detecting plasma NETs is through ELISA using a single NET-associated capture antibody such as anti-MPO, anti-NE or anti-CitH3, combined with anti-DNA antibodies for detection (30). First, we tested different capture antibody concentrations of 1µg/mL, 2.5µg/mL, 5µg/mL, and 10µg/mL for anti-NE (SantaCruz), anti-MPO (Bio-Rad), and anti-CitH3 (Abcam) antibodies followed by the assessment of two developing substrates commonly used in the literature– TMB (3,3’,5,5’-tetramethylbenzidine) (**Figure S1**) and ABTS (2’-azinobis 3-ethylbenzothiazoline-6-sulfonic-acid 2’-azinobis 3-ethylbenzothiazoline-6-sulfonic-acid) (**Figure S2**). We found the optimal concentration of capture antibody to be 5µg/mL and TMB was the superior developing substrate due to its higher sensitivity and shorter reaction time.

**Figure 2:**
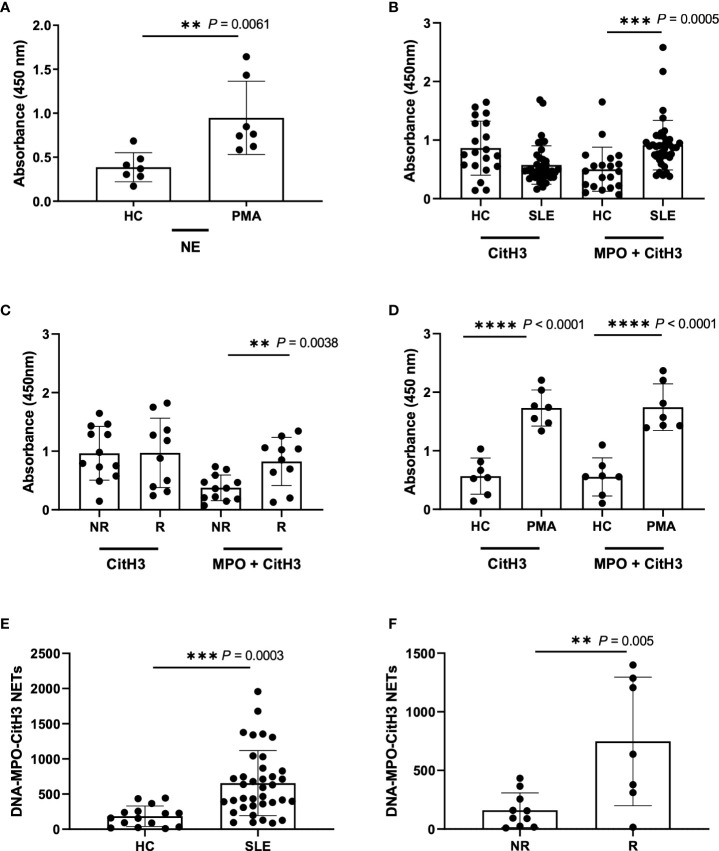
Detection of NETs using different ELISA methodologies with plasma samples from Healthy donors, PMA stimulated donors, SLE patient, and non-risk and risk GaP patient samples. **(A)** ELISA plate coated with 5µg/ml NE (SantaCruz) antibody using Healthy (HC) and PMA stimulated plasma samples (n=7). **(B)** ELISA plates were coated with CitH3 antibody (Abcam) or combination of MPO (Abcam) and CitH3 (Abcam) antibodies at a concentration of 5µg/ml to test healthy donors (n=20) against SLE patient donors (n=40), **(C)** non-risk (n=12) and risk (n=10) associated samples from the Genotype and Phenotype registry, and **(D)** healthy donor against PMA stimulated plasma samples (n=7). ELISA was blocked using additional 5%NRS in the buffer and developed using TMB substrate and results demonstrate differences in plate absorbance values in the presence and absence of MPO (Abcam) in the initial antibody coating **(B-D)**. **(E)** Extrapolated data from standard curve to represent DNA-MPO-CitH3 NET content in healthy controls (n=15) and SLE (n=37) and **(F)** GaP non-risk (n=10) and risk (n=7) samples using data from MPO+CitH3+DNA ELISA. Single data points represent individual donors. Plotted data are after background subtraction. Data are presented as mean ± SD. P values are reported after unpaired parametric T test was performed. **<0.01; ***<0.001; ****<0.0001.

As single capture antibodies, only anti-NE provided a consistent detection of increased circulating NETs in PMA-stimulated samples as compared to unstimulated healthy controls (**Figures S1A–D**, **Figure 2A**). Antibodies directed against MPO or CitH3 gave higher background in all tested concentrations (**Figures S1E–L**). In an attempt to lower background with these two antibodies, experiments were repeated with the addition of 5% Normal Rat Serum (NRS) to the blocking buffer; the background was reduced, and sensitivity of NET detection was increased for both antibodies, but only anti-CitH3 antibody provided a significant difference between PMA-stimulated and unstimulated samples (**Figures S3A, B**). Given that anti-MPO antibodies from BioRad still showed low sensitivity for NETs, we tested another commonly used anti-MPO antibody (Abcam cat#25989) (34, 35) and found the expected significant increase in PMA-induced NETs (**Figure S3C**). Interestingly, when re-testing anti-NE antibody with NRS blocking buffer, trends were like those shown in **Figures S1A-D**, however we found an overall reduction in NET detection (**Figures S3D–G**). Thus, 5µg/mL anti-NE (SantaCruz) antibody without the addition of 5% NRS to blocking buffer, and 5µg/mL anti-MPO (Abcam) or anti-CitH3 (Abcam) antibody with the addition of 5% NRS provided the optimal conditions for NET detection by single antibody capture ELISA.

We next asked the question of whether we could increase specificity and/or sensitivity for plasma NETs by multiplex ELISA using the combination of all three antibodies for capture. While we detected a significant increase in NET detection between the unstimulated healthy control and PMA-stimulated samples using 5 µg/mL anti-NE (SantaCruz), -MPO (Abcam) and -CitH3 (Abcam) together, it was not to a higher level than that detected by 5 µg/mL anti-NE or -CitH3 alone (**Figure S1C**, **Figures S3A, H**). This was likely due to the requirement of 5% NRS in blocking buffer for anti-MPO and -CitH3 that reduces the specificity of anti-NE antibody (**Figures S3D–G**). Hence, we tried the combination of anti-MPO (Abcam) and -CitH3 (Abcam) antibodies in 5% NRS blocking buffer and detected a significant and striking increase in NET detection from SLE patient and *IRF5* risk plasma samples (**Figures 2B, C**), but not from PMA-induced NETs, when comparing anti-CitH3 alone and combined anti-MPO + anti-CitH3 antibody detection (**Figure 2D**) (32).

Last, we generated an in-house standard of known DNA concentration to quantify plasma NETs by standard curve extrapolation of SLE and GaP samples in both anti-CitH3, and anti-MPO + -CitH3 ELISA (**Figures S4A, B**). The standard curve from combined anti-MPO + -CitH3 antibodies had a higher linear dynamic range than anti-CitH3 antibody alone, allowing the NET content in samples with higher absorbances to be accurately determined (**Figures S4A, B**, **Figures 2E, F**). Therefore, we did not detect significant differences in DNA-CitH3 NET content between SLE samples and healthy controls or homozygous risk and non-risk samples when extrapolating data from the anti-CitH3 standard curve (**Figures S4C, D**). However, the calculated DNA-MPO-CitH3 NET content in SLE samples consistently showed a significant increase over healthy controls (**Figure 2E**), and similar significant differences were seen between homozygous risk and non-risk donors (**Figure 2F**).

### Plasma NET smear assay

**Figure 3:**
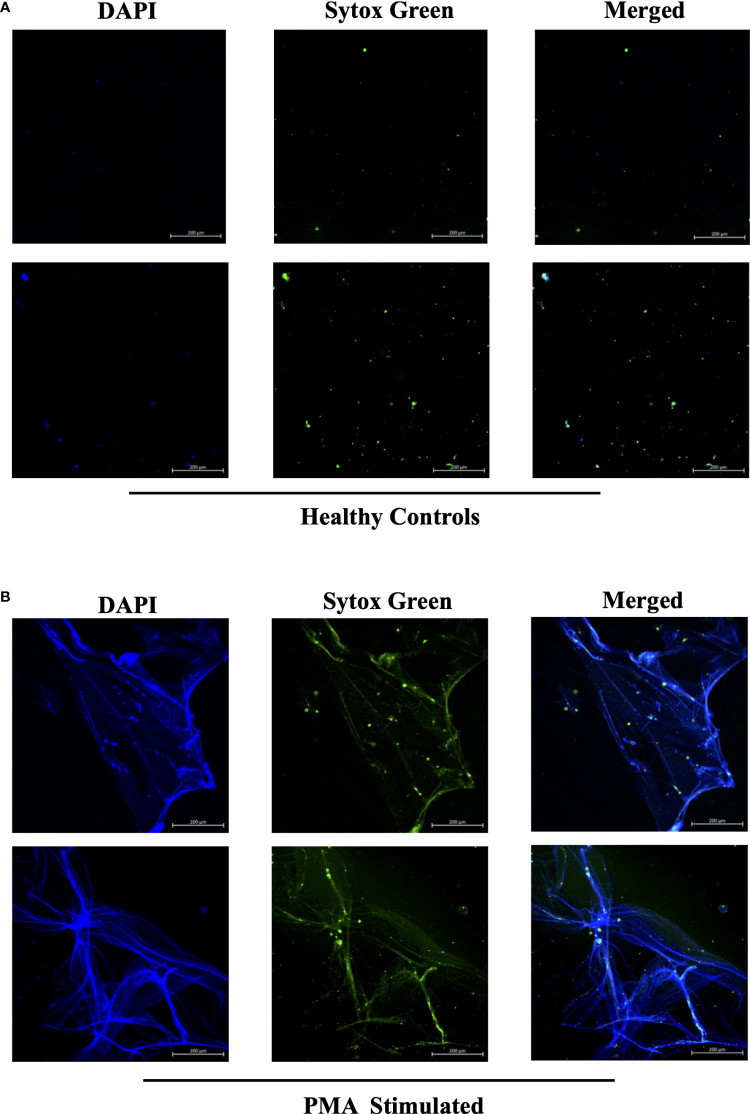
Sytox Green and DAPI staining of PMA stimulated and unstimulated samples to confirm presence of circulating NETs. Plasma smeared poly-l-lysine slides were prepared and stained with Sytox Green and DAPI for **(A)** unstimulated healthy controls and **(B)** PMA stimulated controls. Overlap of DAPI and Sytox Green channels confirm presence of circulating NETs in PMA stimulated donors. Individual merged images represent separate donors (n=4). All images were taken on ZEISS Confocal M880 at 20x objective. Set scale 200µm.

**Figure 4:**
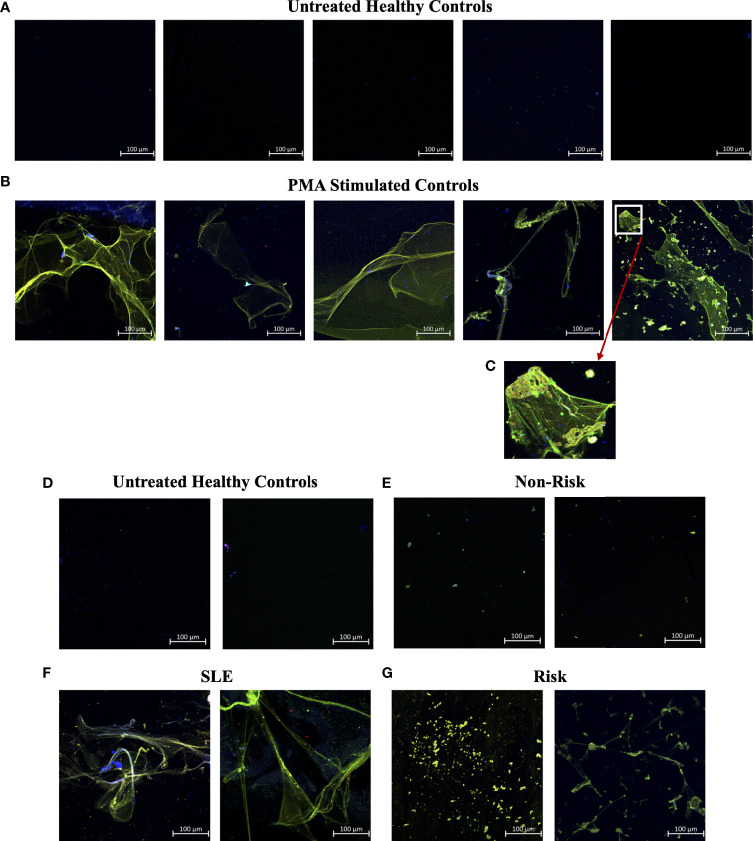
Smear assay showing higher NETs in plasma from PMA stimulated samples, SLE samples, and risk GaP patient samples when compared to healthy donors. Representative images of plasma smeared poly-l-lysine slides stained with MPO (green, AF488), CitH3 (red, AF594) and DAPI (Blue). **(A)** Untreated healthy plasma smears display lower concentration of circulating NETs than **(B)** plasma of PMA stimulated samples. **(C)** Last image of PMA stimulated sample zoomed in 5x to show differential staining of MPO (green) and CitH3 (red) antibodies. **(D)** Healthy donor and **(E)** non-risk donor samples also show lower concentration of circulating NETs than **(F)** plasma of SLE and **(G)** *IRF5* homozygous risk donors. Individual merged images represent separate donors (n=18). All images were taken on ZEISS Confocal M880 at 20x objective. Set scale 100µm.

**Figure 5:**
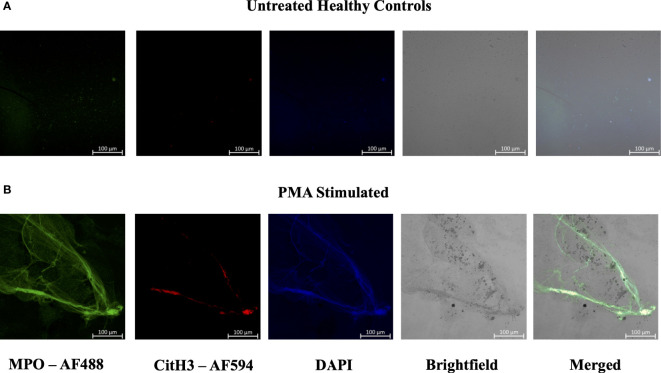
Plasma smear circulating NETs can be detected through brightfield microscopy. Plasma smeared poly-l-lysine slides were prepared and stained with MPO antibody (green, AF488), CitH3 (red, AF594) and DAPI (blue) and single channel images were obtained for **(A)** unstimulated healthy control and **(B)** PMA stimulated samples. NETs were identified and brightfield images overlapped with specific antibody markers in **(B)** PMA stimulated sample, therefore confirming the presence of circulating NETs. Individual merged images represent separate donors (n=2). All images were taken on ZEISS Confocal M880 at 20x objective. Set scale 100µm.

**Figure 6:**
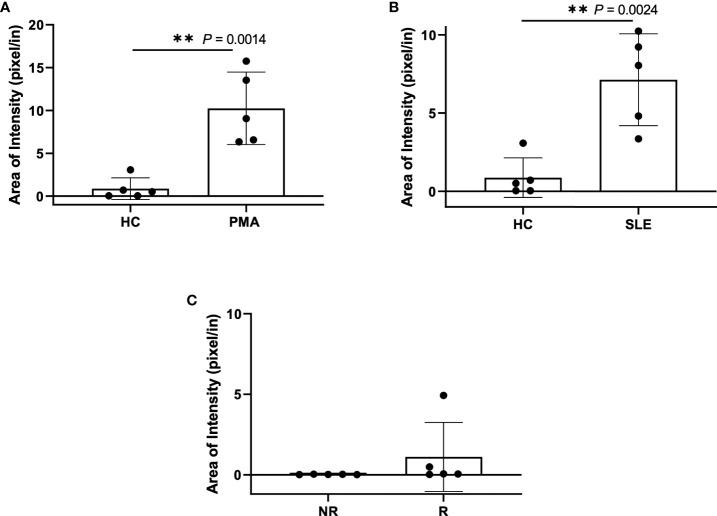
Plasma smear quantification using 20x objective and average threshold-area pixel intensity. Using ImageJ, representative plasma smear images were converted to 8-bit grayscale images and pixel intensity threshold range was set from 40-255. Images were measured and averages of threshold-area pixel intensity were computed for **(A)** healthy control and PMA stimulated (n=5), **(B)** healthy control and SLE (n=5), and **(C)** GaP risk and non-risk (n=5). Observed differences between sample groups correlate to previously calculated NET quantity in ELISA. Data are presented as mean ± SD. P values are reported after unpaired parametric T test was performed. **<0.01.

**Figure 7:**
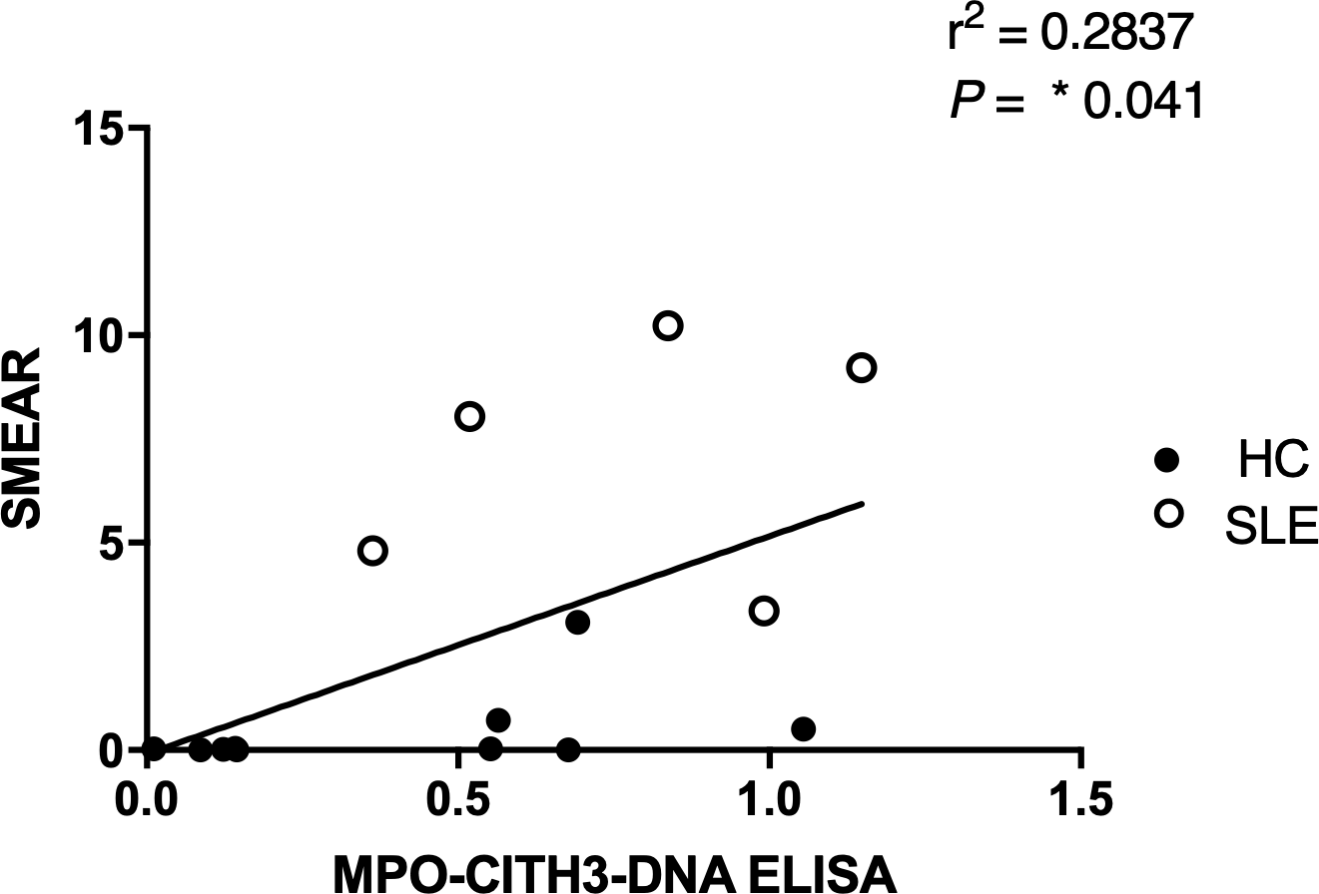
Correlation analysis between MPO + CitH3 + DNA ELISA and plasma smear assay across patient samples. Quantity of circulating NETs in healthy control (n = 9) and SLE (n=5) patient samples were measured using the two different assays, and the correlation between assay results was determined. Single data points represent individual donors. Two-tailed correlation analysis between data sets was calculated using Pearson correlation coefficients assuming Gaussian distribution in GraphPad Prism 8. R^2^ and p-value of linear regression are reported above.

In efforts to develop a NET assay that is quantitative, relatively quick, inexpensive, and utilizes smaller volumes of patient plasma, we designed the NET smear assay. As before, we first optimized the assay using Sytox Green and DAPI to detect plasma DNA. 1 µl of PMA-stimulated or unstimulated plasma was fixed onto poly-L-lysine glass slides, followed by staining with Sytox Green and DAPI. Representative images in **Figure 3** confirm the presence of plasma NETs. We then utilized anti-MPO(Abcam) and-CitH3(Abcam) primary antibodies, followed by secondary fluorescent staining, along with DAPI, to visually assess NET architecture. Unstimulated healthy control samples showed minimal staining by anti-MPO (green), anti-CitH3 (red) and DAPI (blue) (**Figure 4A**), while PMA-stimulated samples showed a range of NET architectures with positive staining by both antibodies and DAPI (**Figure 4B**). Anti-MPO and -CitH3 antibodies successfully displayed specific differential staining patterns within the structures of circulating NETs (**Figure 4C**). Given that the same plasma samples were used for both ELISA and smear assay, these results visually confirm the presence of plasma NETs. Indeed, we can identify more NETs in plasma from SLE patients and risk donors as compared to unstimulated healthy control and non-risk samples, respectively (**Figures 4D-G**). Surprisingly, NETs could be visualized under brightfield directly after smearing and fixing, providing the same results as immunofluorescence staining (**Figure 5**). Moreover, quantification of 20X images using ImageJ provided a higher average intensity of the threshold area in PMA-stimulated samples compared to unstimulated samples (**Figure 6A**). While similar differences were found in SLE samples as compared to unstimulated healthy controls (**Figure 6B**), no significant change between risk and non-risk samples was found (**Figure 6C**). Albeit the average threshold area of NETs was higher in risk versus non-risk samples. Since this quantification was performed on 20X images in an area showing NETs, we performed the same quantification on 2X images that are non-subjective and cover most of the plasma-smeared area and obtained similar results (**Figure S5**).To compare the quantitative results of our newly developed smear assay with data obtained from DNA-MPO-CitH3 ELISA, a two-tailed correlation analysis was computed using matched data across healthy and SLE patient samples (**Figure 7**). The correlation between the two assays was found to be positive and significant.

## Discussion

In the present study, we optimized a multiplex ELISA that provided increased sensitivity, with a wider range of detection of plasma NETs as compared to single antibody capture (**Figure 2**). Moreover, we developed a relatively quick and simple, quantitative NET smear assay that displays significant, positive correlation with ELISA results (**Figure 7**). Both methods were able to consistently detect differences in plasma NET levels between patient samples – healthy PMA-stimulated vs. unstimulated, SLE vs. healthy controls, and homozygous *IRF5*-SLE risk vs. non-risk. To the best of our knowledge, this is the first study to visualize and quantify NET levels in only 1 ul of patient plasma. Notably, the method requires minimal time, reagents, specialized equipment and/or costs (22, 34, 36–38).

There are several diseases that associate with elevated serum or plasma NET levels, including SLE, diabetes, COVID-19, and cardiovascular disease, in which the NETs are also thought to participate in disease pathogenesis (3). In many autoimmune diseases, NETs are considered a source of autoantigen leading to increased type I IFN production and autoantibodies (6, 8, 25, 39). This is supported by the finding of mutations in *DNAseIL3* in SLE patients that results in increased circulating NETs (40, 41). High levels of NETs have also been detected in the blood of patients with COVID-19 (17–19). In COVID-19, elevated circulating neutrophils are associated with and predict severe respiratory disease and unfavorable outcomes (42, 43). Moreover, COVID-19 patients who developed thrombosis were found to have more circulating NETs in their blood compared to patients without clinical thrombosis, associating them with a higher risk of developing the condition (42). Higher levels of NETs have also been reported in diabetes, which impair atherosclerosis resolution by increasing plaque inflammation (14). Indeed, increased NET levels in diabetic patients were found to contribute to increased cardiovascular disease risk (14), and NETs in cardiovascular disease are known to interfere with the activation of coagulation pathways (44).

Given that NETosis is a central mechanism coordinating the innate immune response and NETs serve as biomarkers and/or risk factors of many inflammatory and autoimmune diseases (39, 42, 45), there is a strong need in the field to further optimize and develop new methods of NET detection in patient plasma or serum. In the case of COVID-19, timely detection of even low quantities of circulating NETs may change a patient’s treatment regimen and outcome (18). We used plasma from SLE patients with active or inactive disease, as recently described (25), and from healthy donors that carry either the homozygous *IRF5*-SLE haplotype that is a risk factor for SLE, or the homozygous non-risk haplotype, to assess the sensitivity and range of plasma NET detection. Use of the anti-DNA antibody along with 5 µg/ml anti-CitH3 (Abcam) as capture antibody provided the most sensitive single antibody ELISA for plasma NET detection. The sensitivity of detection was further improved in patient samples with the combination of anti-MPO(Abcam) and -CitH3(Abcam) antibodies plus 5% NRS in blocking buffer (**Figures 2B–D**). Differences in NET levels extrapolated from the standard curve further confirmed the accuracy and sensitivity of the combined MPO + CitH3 ELISA, as the standard curve calculated from CitH3 alone did not cover the data range. Notably, for the NET smear assay, 1 ul plasma stained with Sytox Green and DAPI for 15 min provided clear NET structures (**Figure 3**) that correlated with brightfield images taken immediately after smearing (**Figure 5**). Like immunofluorescence staining of neutrophils on poly-L-lysine-coated plates (32, 38), specificity for NETs was increased by staining with anti-MPO(Abcam) and -CitH3(Abcam) antibodies along with DAPI (**Figure 4**). While data from both methods of NET detection revealed significant and comparable differences between healthy donor and SLE patient plasma, results from healthy donor GaP samples indicate that the ELISA is more sensitive and has a wider range for detecting low levels of plasma NETs, than smear assay. Nevertheless, we found absolute congruence and correlation between the two methods supporting the utility of the NET smear assay for rapid visualization of plasma NETs (**Figure 7**). In the past decade, other immune cells have been shown to produce extracellular traps. For example, macrophages undergo METosis making macrophage extracellular traps, and eosinophils have been reported to produce eosinophil extracellular traps (46, 47). Our newly developed immunofluorescence smear assay for the detection of NETs therefore has the potential to be extended to the detection of ETosis using cell type-specific antibodies. Moreover, different pathways/mechanisms of NETosis may be induced depending on the trigger that leads to differential NET composition (48, 49). Thus, our methods may be extended to study different mechanisms of NETosis by selecting appropriate antibodies, such as inclusion of anti-PAD4 antibodies.

Last, disparity in the two methods became evident when using fresh versus frozen plasma samples. An advantage of the ELISA is it can detect circulating NETs effectively from plasma samples stored at -80° C for long periods of time (> 6 months), as it can detect both intact and fragmented NETs. By smear assay, we found that plasma NET structures were generally maintained when stored at -80° C for up to 6 months, yet after 6 months, only ‘chewed’ NET fragments could be visualized that compromised their quantification, as seen in GaP samples in **Figure 4G**. Preliminary studies suggest that adding EDTA/EGTA immediately after plasma isolation will prolong the stability of intact NET structures for quantification.

## Data availability statement

The original contributions presented in the study are included in the article/**supplementary material**. Further inquiries can be directed to the corresponding author.

## Ethics statement

The studies involving human participants were reviewed and approved by Feinstein Institutes for Medical Research IRB. The patients/participants provided their written informed consent to participate in this study.

## Author contributions

BM and JB performed the ELISA and NET smear assay. BM and BB designed the research and supervised the project. BM, JB, and BB wrote the manuscript. All authors contributed to the article and approved the submitted version.

## Funding

This work was supported by grants from the National Institutes of Health NIAMS 1 R01 AR 076242-03, Department of Defense CDMRP LRP W81XWH-18-1-0674, and Lupus Research Alliance to BB.

## Acknowledgments

We thank members of the GaP Registry and G. Klein, M. DeFranco, and K.M. Elmaliki for consenting and scheduling donors. This work was supported by grants from the National Institutes of Health NIAMS 1 R01 AR 076242-03, Department of Defense CDMRP LRP W81XWH-18-1-0674, and Lupus Research Alliance to BJB. We would also like to thank members of “The NETwork to Target Neutrophils in COVID-19” for inspiration to develop new methodologies to detect NETosis.

## Conflict of interest

The authors declare that the research was conducted in the absence of any commercial or financial relationships that could be construed as a potential conflict of interest.

## Publisher’s note

All claims expressed in this article are solely those of the authors and do not necessarily represent those of their affiliated organizations, or those of the publisher, the editors and the reviewers. Any product that may be evaluated in this article, or claim that may be made by its manufacturer, is not guaranteed or endorsed by the publisher.
