# Supplementary material for: Detection of neutrophil extracellular traps in patient plasma: method development and validation in systemic lupus erythematosus and healthy donors that carry IRF5 genetic risk
Source: Front Immunol. 2022 Jul 26;13:951254. doi: 10.3389/fimmu.2022.951254 (PMC9360330; doi:10.3389/fimmu.2022.951254)
Supplement: Supplementary file 1 [file DataSheet_1.docx]

**Supplemental Material**

**NE**

**(C)**

**(D)**

**(A)**

**(B)**

**MPO**

**(E)**

**(H)**

**(G)**

**(F)**

**CitH3**

**(I)**

**(L)**

**(K)**

**(J)**

**10µg/ml**

**5µg/ml**

**2.5µg/ml**

**1µg/ml**

**Figure S1** Comparing antibody coating concentration using TMB as developing substrate on healthy control and PMA stimulated plasma samples in ELISA. ELISA experiments using (A, E, I) 1µg/ml, (B, F, J) 2.5µg/ml, (C, G, K) 5µg/ml, and (D, H, L) 10µg/ml of coating antibody. NE (SantaCruz) antibody (A-D), MPO (BioRad) antibody (E-H), and CitH3 (Abcam) antibody (I-L) experiments were blocked with 5%BSA and developed using TMB substrate. All experiments show a comparison between healthy control (HC) and PMA stimulated samples (n=6). Single data points represent individual donors. Plotted data are after background subtraction. Data are presented as mean ±SD. P values are reported after unpaired parametric T test was performed.

**(D)**

**(C)**

**(B)**

**(A)**

**NE**

**MPO**

**(H)**

**(G)**

**(F)**

**(E)**

**CitH3**

**(L)**

**(K)**

**(J)**

**(I)**

**10µg/ml**

**5µg/ml**

**1µg/ml**

**2.5µg/ml**

**Figure S2** Comparing antibody coating concentration using ABTS as developing substrate on healthy control and PMA stimulated plasma samples in ELISA. ELISA experiments using (A, E, I) 1µg/ml, (B, F, J) 2.5µg/ml, (C, G, K) 5µg/ml, and (D, H, L) 10µg/ml of coating antibody. NE (SantaCruz) antibody (A-D), MPO (BioRad) antibody (E-H), and CitH3 (Abcam) antibody (I-L) experiments were blocked with 5%BSA and developed using ABTS substrate. All experiments show a comparison between healthy control (HC) and PMA stimulated samples (n=6). Single data points represent individual donors. Plotted data are after background subtraction. Data are presented as mean ±SD. P values are reported after unpaired parametric T test was performed.

**(B)**

**(A)**

**(H)**

**(D)**

**(G)**

**(C)**

**(E)**

**(F)**

**Figure S3** Effects of the addition of 5% Normal Rat Serum (NRS) to 5% BSA Blocking buffer on NE, MPO and CitH3 ELISA experiments using healthy control and PMA stimulated samples. Altered blocking buffer with 5% NRS shows improved results in ELISA experiments using (A) 5µg/ml of CitH3 (Abcam) antibody, (B) MPO (BioRad) antibody, and (C) MPO (Abcam) antibody. Comparative ELISA results with NE (SantaCruz) antibody and altered blocking buffer show decrease in average absorbance at (D) 1µg/ml, (E) 2.5µg/ml, (F) 5µg/ml, and (G) 10µg/ml. (H) Reduced ELISA values are also seen when combining NE (SC), MPO (Abcam) and CitH3 (Abcam) coating antibodies together at 5µg/ml using altered NRS block. All experiments were developed using TMB substrate. Results show a comparison between healthy control (HC) and PMA stimulated samples (n=6). Single data points represent individual donors. Plotted data are after background subtraction. Data are presented as mean ±SD. P values are reported after unpaired parametric T test was performed.

**(C)**

**(D)**

**(B)**

**(A)**

**Figure S4** Standard curve for MPO-CitH3-DNA ELISA compared to standard curve for CitH3-DNA ELISA shows better spread and representation of data. (A) Four parameter logistic curve for standard from MPO-CitH3-DNA ELISA and (B) CitH3-DNA ELISA. Curves were computed and graphed using online program MyAssays. Standard was prepared by 2x dilution of combined plasma from three healthy donors after stimulation with PMA. (C) Data extrapolated from CitH3 (Abcam) single-coating ELISA standard curve for healthy control (n=10) and SLE samples (n=36), and (D) non-risk (n=5) and risk (n=5) samples. Single data points represent individualdonors. Plotted data are after background subtraction. Data are presented as mean ±SD.

**(A)**

**(B)**

**(C)**

**Figure S5** Quantification of plasma smear using 2x objective to validate 20x objective calculations. Using ImageJ, plasma smear images were converted to 8-bit grayscale images and pixel intensity threshold range was set from 40-255. Images were measured and averages of threshold-area pixel intensity were computed for (A) healthy control and PMA stimulated (n=5), (B) healthy control and SLE (n=5), and (C) GaP risk and non-risk plasma samples (n=5). Observed differences between sample groups correspond to previously calculated differences in 20x sample group. Single data points represent individual donors. Data are presented as mean ±SD. P values are reported after unpaired parametric T test was performed.
